# Editorial: *Ralstonia solanacearum*–Plant Interactions: Plant Defense Responses, Virulence Mechanisms and Signaling Pathways

**DOI:** 10.3389/fpls.2022.890877

**Published:** 2022-05-05

**Authors:** Yasufumi Hikichi, Marc Valls, Stephane Genin

**Affiliations:** ^1^Faculty of Agriculture and Marine Science, Kochi University, Kochi, Japan; ^2^Genetics Section, Universitat de Barcelona, Catalonia, Spain; ^3^LIPME, Université de Toulouse, INRAE, CNRS, Castanet-Tolosan, France

**Keywords:** *Ralstonia solanacearum*-plant interactions, plant defense responses, virulence mechanisms, signaling pathways, development of the disease control system

In the Research Topic *Ralstonia solanacearum–Plant Interactions: Plant Defense Responses, Virulence Mechanisms and Signaling Pathways*, we aimed to collect manuscripts dealing with not only molecular mechanisms of *R. solanacearum* virulence and host responses but also development of the disease control system such as the disease-resistance cultivars and microbial inoculants.

The molecular study of the interaction of strains of the *R. solanacearum* with their host plants has largely focused on solanaceous species, mainly on tomato and to a lesser extent potato. Nevertheless, there are plants of major agronomic interest outside the Solanaceae on which researchers are now seeking to better understand the mechanisms of susceptibility/resistance to bacterial wilt. In their study, Tan et al. make a valuable contribution toward the understanding of the virulence mechanisms of strains pathogenic on legume plants, which have so far been relatively unexplored. By screening more than 100 peanut cultivars, Tan et al. were able to identify two strains that were phylogenetically very close, but differed remarkably in their virulence profile. The article presents the genome analysis of these strains and examines the major differences observed. Several candidate genes to explain the difference in virulence were identified, including regulatory genes, genes contributing to motility and adhesion, and only four type 3 effectors differing in the repertoires of these two strains.

In addition to genomic or transcriptomic comparison studies, more advanced approaches aimed at better quantification of proteins or metabolites are now routinely used. Yang et al. conducted a comparative study to identify differences in metabolite content in tobacco xylem sap during *R. solanacearum* infection, both in susceptible and resistant tobacco cultivars. Interestingly, among the metabolites differentially concentrated, several of them appear to be significantly induced in the susceptible cultivar and not in the resistant one, which opens up interesting avenues for future investigation of how the pathogen induces such metabolic reprogramming in its host.

The article by Pan et al. addresses tobacco gene expression in the highly resistant 4411-3 and the moderately resistant K326 varieties in basal conditions or at three time points after *R. solanacearum* inoculation. This work revealed an unusually large number of genes induced by the pathogen. Previous transcriptomic and functional studies in other plant hosts had shown that cell wall components and plant hormones including auxin and ABA were involved in the responses to *R. solanacearum*. Pan et al. confirmed these results in tobacco, and found that the levels of both hormones are lower in plants showing higher resistance to the disease. The study also identified response specificities and suggested the involvement of ABC transporters, endocytosis and the metabolism of glutathione and glycerolipids in tobacco resistance against *R. solanacearum*. The study provides a number of candidate genes related to cell wall metabolism, plant hormone and glutathione metabolism whose involvement in *Nicotiana* resistance to *R. solanacearum* deserves to be further studied.

Plant cells under pathogen attack often induce the formation of Reactive-Oxygen Species (ROS) that trigger a signaling cascade, increase cell wall cross-linking, trigger plant defense and act as antimicrobial agents. Plant-associated bacteria use oxidative stress response genes to counter-attack ROS. The work by Tondo et al. characterized an *R. solanacearum* monofunctional heme catalase named KatE, a ROS-scavenging enzyme long considered a key candidate virulence gene. The authors demonstrated that *katE* expression is induced upon hydrogen peroxide treatment and transcriptionally activated by the central virulence regulator HrpG. However, although KatE was shown to be essential for bacterial survival to oxidative stress, it was proven that it does not contribute to *R. solanacearum* virulence or bacterial growth in the plant. This result is surprising, considering that the gene was proposed in a previous study as essential for survival *in planta* and makes us wonder whether oxidizing conditions are important in other environments encountered by *R. solanacearum* during its life cycle such as the phylosphere or the rhizosphere.

More than 100 type III effectors (T3Es) are predicted among different *R. solanacearum* strains, with an average of about 70 T3Es in each strain. Among them, 32 core T3Es are found to be conserved among the RSSC. Highly variable repertoires of T3Es might be responsible for the host range of different *R. solanacearum* strains. Lei et al. created the 42 T3 effector (all 21 T3Es of AWR, GALA, HLK, and SKWP families, 15 core T3Es, and six extended core T3Es) genes-deletion mutant from the strain OE1-1, which are virulent on both tobacco and eggplant plants. The mutant exhibited a significantly reduced colonization in both tobacco and eggplant plants and a significantly reduced virulence on them, demonstrating that all the core and extended core T3Es are nearly crucial for virulence of the strain OE1-1 toward host plants, and the T3Es-free strain will enable primary function of individual T3Es in host cells.

T3Es are common elicitors of intracellular plant immune receptors encoded by nucleotide-binding domain and leucine-rich repeat containing (NLR) genes. T3Es contribute to both virulence on a susceptible host and an immune response on non-host plants, which have the cognate receptor to recognize the T3E. NLR genes typically confer strong, dominant resistance to pathogens that deliver the cognate recognized T3E. The NLR protein Roq1 of *Nicotiana benthamiana* recognizes the T3Es XopQ and HopQ1, which are highly conserved and present in most plant pathogenic strains of *Xanthomonas* and *Pseudomonas syringae*. Furthermore, A homolog of XopQ/HopQ1, RipB, is present in most *Ralstonia* strains. Thomas et al. found that expression of *Roq1* in tomato plants confers immunity to *Xanthomonas, P. syringae*, and *Ralstonia* upon recognition of the cognate pathogen T3E, demonstrating the widespread potential of using naturally occurring plant immune receptors to manage diverse and difficult to control pathogen species.

The studies collected in this Research Topic will shed light on a deeper understand of *R. solanacearum*-Plant Interactions and ultimately pave the way to deal with this devastating pathogen and developing sustainable control system on the disease.

## Author Contributions

All authors listed have made a substantial, direct, and intellectual contribution to the work and approved it for publication.

## Conflict of Interest

The authors declare that the research was conducted in the absence of any commercial or financial relationships that could be construed as a potential conflict of interest.

## Publisher's Note

All claims expressed in this article are solely those of the authors and do not necessarily represent those of their affiliated organizations, or those of the publisher, the editors and the reviewers. Any product that may be evaluated in this article, or claim that may be made by its manufacturer, is not guaranteed or endorsed by the publisher.

